# Lipid abnormalities in patients with Rheumatoid Arthritis

**DOI:** 10.12669/pjms.331.11699

**Published:** 2017

**Authors:** Uzma Erum, Tasnim Ahsan, Danish Khowaja

**Affiliations:** 1 Dr. Uzma Erum, MBBS, FCPS Trainee. Medical Unit-II, Rafiqee Shaheed Road, Jinnah Postgraduate Medical Centre, Karachi, Pakistan; 2 Prof. Tasnim Ahsan, MRCP, FRCP, FRCP, FRCP. Medical Unit-II, Rafiqee Shaheed Road, Jinnah Postgraduate Medical Centre, Karachi, Pakistan; 3 Dr. Danish Khowaja, MBBS, FCPS Trainee. Medical Unit-II, Rafiqee Shaheed Road, Jinnah Postgraduate Medical Centre, Karachi, Pakistan

**Keywords:** Cholesterol, Dyslipidemia, Rheumatoid Arthritis

## Abstract

**Objective:**

To determine the frequency of dyslipidemia in patients with Rheumatoid Arthritis.

**Methods:**

This is a prospective, cross-sectional, observational study, conducted at the ‘Rheumatology Clinic’ of Jinnah Postgraduate Medical Center (JPMC), Karachi, from November 2013 to May 2014. A total of 200 patients of Rheumatoid Arthritis (RA), diagnosed according to the ACR/EULAR criteria 2010, were included in the study. Laboratory investigations including creatinine, ALT, CBC, TSH and fasting lipid profile (LDL, HDL, and Total cholesterol) were done for all patients.

**Results:**

Out of 200 patients, 23 (11.5%) were male and 177 (88.5%) were female. The mean age was 36.31±10.46 years and the mean duration of disease was 3.82±3.03 years. A total of 107 (53.5%) patients had dyslipidemia, and the commonest abnormality was a low HDL, seen in 83 (41.5 %) patients.

**Conclusion:**

Dyslipidemia was frequently observed in Rheumatoid Arthritis. This may be considered as a secondary impact of chronic inflammatory state, seen in RA. Lipid abnormalities should be sought at regular intervals, and corrective actions taken to mitigate increased cardiovascular disease risk.

## INTRODUCTION

Rheumatoid Arthritis (RA) is a chronic multisystem disease with heterogeneous presentation, variable disease progression and extra-articular manifestations. The estimated prevalence of RA has been reported from Northern Pakistan as 0.55%;^1^ while, the prevalence of RA in adult Indian population is reportedly 0.75%.^2^ It has been observed that RA patients have greater rates of cardiovascular (CV) morbidity and mortality.^3^,^4^ Studies indicate that the risk of incidental cardiovascular disease (CVD) is increased by 48% in patients with RA compared to the general population.^5^

Lipid metabolism is a complex process, especially when associated with chronic inflammatory states; therefore in many autoimmune diseases lipid abnormalities are frequently seen. The involvement of common pro-inflammatory cytokines, such as interleukin - 1 and 6, tumor necrosis factor-alpha (TNF-α), play a role in the development and progression of both RA and atherosclerosis. Variable degrees of heightened inflammatory state is seen in RA, which alters the properties of HDL cholesterol, hence, the HDL paradoxically assumes pro-inflammatory properties, thereby accelerating endothelial dysfunction and plaque formation.^6^

Many studies have previously reported lipid abnormalities in RA patients.^7^-^9^ The dyslipidemia observed in RA patients is paradoxical to the general population, comprising of low total cholesterol and low HDL.^10^,^11^ The disproportionate ratio between HDL and TC levels, results in an increased atherogenic index (TC:HDL ratio), which is an important prognostic marker for cardiovascular disease (CVD). Dyslipidemia is prevalent in our general population as well. Therefore, this study was designed to observe the pattern of abnormal lipid profile in relation to chronic inflammatory state seen in RA patients.

## METHODS

### Study Design

This was a cross-sectional observational study, conducted at the ‘Rheumatology Clinic’ of JPMC, Karachi, from November 2013 to May 2014.

### Inclusion Criteria

A total of 200 patients with the diagnosis of RA of > 6 month duration, according to the ACR/EULAR criteria 2010,^12^ of either sex, between the ages of 20-60 years were included in the study.

### Exclusion Criteria

Patients not consenting and those with chronic diseases like Diabetes Mellitus, Hypothyroidism, Chronic kidney disease, Chronic liver disease, smokers, patients taking oral contraceptive pills and statins were excluded.

### Dyslipidemia

A total cholesterol of ≥200 mg/dl or HDL Cholesterol <40 mg/dlor LDL Cholesterol ≥160 mg/dl were used to define the cut-off values for dyslipidemia, according to the National Cholesterol Education Program- Adult Treatment Panel III (NCEP-ATP III) guidelines.^13^

### Data Collection

All the patients who fulfilled the inclusion criteria were included in the study. Prior approval was obtained from the Institutional Ethical Review Committee. Informed consent was taken from all the patients before assigning them to the study. History and physical examination was recorded in all patients. Blood sample was collected in a sterile manner after an overnight fast for serum cholesterol, LDL, and HDL levels. Data was recorded in a pre-designed proforma. Complete blood count (CBC), creatinine, ALT and Thyroid stimulating hormone (TSH) were also checked in all patients.

### Data Analysis

Data was analyzed by using SPSS Version 17. Demographic data was presented as simple descriptive statistics, giving mean and standard deviation for age and duration of disease. For qualitative variables, like gender and dyslipidemia, frequency and percentages were reported.

## RESULTS

**Fig. 1:**
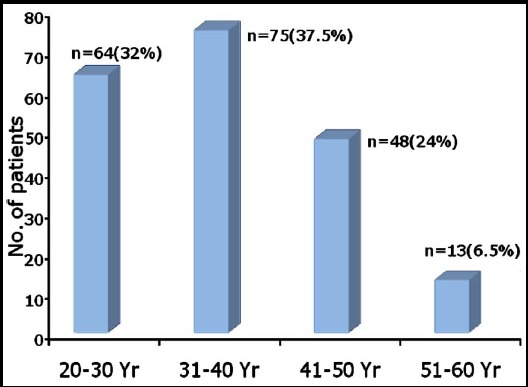
Age distribution among patients with RA.

A total of 200 patients, with the diagnosis of RA, were analyzed; 23 (11.5%) were male and 177 (88.5%) were female. The mean age was 36.31±10.46 and mean duration of disease was 3.82±3.03 years. Most common age bracket was 4^th^ decade of life, constituting 75 (37.5%) patients. (Fig.1). A total of 139 (69.5%) patients had duration of disease of 1-4 years.

**Fig. 2:**
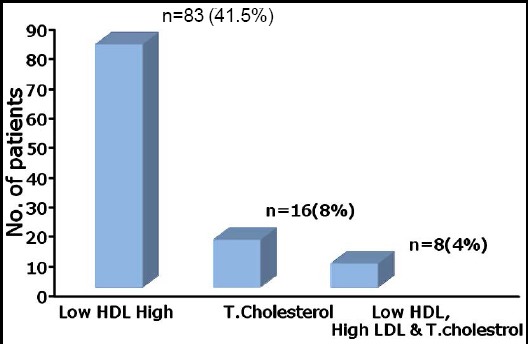
Distribution of lipid fractions in patients with dyslipidemia.

Of all patients, 107 (53.5%) had dyslipidemia. The mean values for total cholesterol, HDL and LDL were 169.68±36.68 mg/dL, 40.02±10.23 mg/dL, and 93.29±26.17 mg/dL respectively (Table-I). Analysis of the lipid fractions in those who had dyslipidemia revealed that 83 patients (41.5%) had low HDL; 16 (8%) had high TC, whereas, 8 (4%) patients had combination of low HDL with high LDL and high TC(Fig.2). Majority of the patients with dyslipidemia were between 20-40 years of age, i.e. 139 (69.5%) patients.

**Table-I:** Lipid levels in RA patients.

	Patients values Means±SD	Max-Min
Total Cholesterol (mg/dl)	169.68±36.68	65-321
HDL (mg/dl)	40.02±10.23	21-64
LDL (mg/dl)	93.29±26.17	54-182

## DISCUSSION

Dyslipidemia (DL) is frequently observed in patients with active RA.^14^ Systemic inflammation has a general effect in lowering circulating lipid levels.^15^ Moreover, patients with RA have an increased CVD risk at relatively low cholesterol levels, in contrast to that observed in the population without RA. These paradoxical changes in the lipid profiles of RA patients are still unclear, and the interactions between lipid fractions, inflammation and CVD risk in RA appears to be very complex. An Indian study, reported38.5% of dyslipidemia in a cohort of RA patients; commonest abnormality being low HDL.^16^ Another study from Pakistan reported an overall derangement of lipid profile in 44.87% of patients with different autoimmune diseases.^17^ The proportion of DL detected in our study is infact a little higher than that already reported in literature, commonest being low HDL. This is in contrast to that, reported by Nisar et al. where the commonest reported abnormality was high cholesterol.^17^ The commonest abnormality of low HDL may be a consequence of the unique pattern of lipid dysfunction that is seen in South-Asian population, which behaves differently in terms of accentuation of CVD risk. No population based study has been reported from Pakistan to reflect the exact prevalence of DL in our population. However, few studies have shown a prevalence of 40-87% of dyslipidemia in the context of metabolic syndrome and diabetes.^18^,^19^ As there is a strong association between metabolic syndrome and diabetes with dyslipidemia, hence it is not possible to relate these results to the entire population.

It has been reported that paradoxical lipid profile renders a patient to three fold increased CVD risk.^20^ Of note is that we need a more stringent control for DL, as ethnic variation in lipid profile is responsible for increased CVD risk and the high inflammatory state in RA patients, as this disease itself accentuates atherosclerosis. Patients with RA might benefit from being targeted with stricter than conventional CVD risk prevention and intervention.^21^,^22^ Evidence suggests that lipid lowering therapy may have beneficial effects in RA by virtue of their potent anti-inflammatory and immune-modulatory properties also.^23^ A vast majority of the patients with DL, in this study, were younger than 40 years, thus excluding aging as an exacerbating factor for occurrence of DL.

Despite there being an increased frequency of dyslipidemia in RA patients, it is unclear as to what extent these changes actually predispose an individual to increased CVD risk.^24^ Other studies have also reported the increased CVD risk even with marginally deranged traditional risk factors in RA patients compared to the healthy population.^25^,^26^

This is a small but systematic study to collate the estimates of lipid abnormalities in RA patients, and the results reflect the need of age-matched controlled comparable trials to observe the lipid profile in RA and control group and its relation, if any, to disease activity. Additional studies are needed to address the value of monitoring lipid profile, and to determine the effect of traditional CVD risk factors and inflammation and their impact on CV outcomes in RA patients. We also need to establish a strategy to monitor lipid profile at regular intervals in every individual with RA.

## CONCLUSION

Dyslipidemia observed in RA is a frequent occurrence and may be considered as a secondary impact of chronic inflammatory state seen in RA patients. Identification and management of DL should be considered an integral part of RA therapeutic strategies to prevent CV morbidity and mortality in RA patients. Further studies are required to determine the interaction between DL and its impact on disease activity in RA patients.
